# [μ-Bis­(diphenyl­phosphino)methane-1:2κ^2^ *P*:*P*′]nonacarbonyl-1κ^3^ *C*,2κ^3^ *C*,3κ^3^ *C*-[tris­(4-methyl­phen­yl)arsine-3κ*As*]-*triangulo*-triruthenium(0)

**DOI:** 10.1107/S1600536809046704

**Published:** 2009-11-21

**Authors:** Omar bin Shawkataly, Imthyaz Ahmed Khan, Chin Sing Yeap, Hoong-Kun Fun

**Affiliations:** a Chemical Sciences Programme, School of Distance Education, Universiti Sains Malaysia, 11800 USM, Penang, Malaysia; b X-ray Crystallography Unit, School of Physics, Universiti Sains Malaysia, 11800 USM, Penang, Malaysia

## Abstract

In the title *triangulo*-triruthenium compound, [Ru_3_(C_21_H_21_As)(C_25_H_22_P_2_)(CO)_9_], the bis­(diphenyl­phos­phino)methane ligand bridges an Ru—Ru bond and the monodentate arsine ligand bonds to the third Ru atom. Both the phosphine and arsine ligands are equatorial with respect to the Ru_3_ triangle. Additionally, each Ru atom carries one equatorial and two axial terminal carbonyl ligands. The three arsine-substituted phenyl rings make dihedral angles of 87.36 (10), 81.96 (10) and 73.37 (11)° with each other. The dihedral angles between the two phenyl rings are 88.08 (12) and 80.15 (10)° for the two diphenyl­phosphino groups. In the crystal packing, the mol­ecules are linked together as dimers *via* inter­molecular C—H⋯O hydrogen bonds. These dimers are stacked down *b* axis. Inter­molecular C—H⋯π and π–π inter­actions [centroid–centroid distance = 3.6383 (13) Å] further stabilize the crystal structure.

## Related literature

For general background to *triangulo-*triruthenium derivatives, see: Bruce *et al.* (1985 ▶, 1988*a* ▶, 1988*b* ▶); Shawkataly *et al.* (1998 ▶, 2009*a* ▶). For related structures, see: Shawkataly *et al.* (2006 ▶, 2009*a* ▶,*b* ▶,*c* ▶). For the synthesis of bis­(diphenyl­phosphino)methane, see: Bruce *et al.* (1983 ▶). For the stability of the temperature controller used for the data collection, see: Cosier & Glazer (1986 ▶).
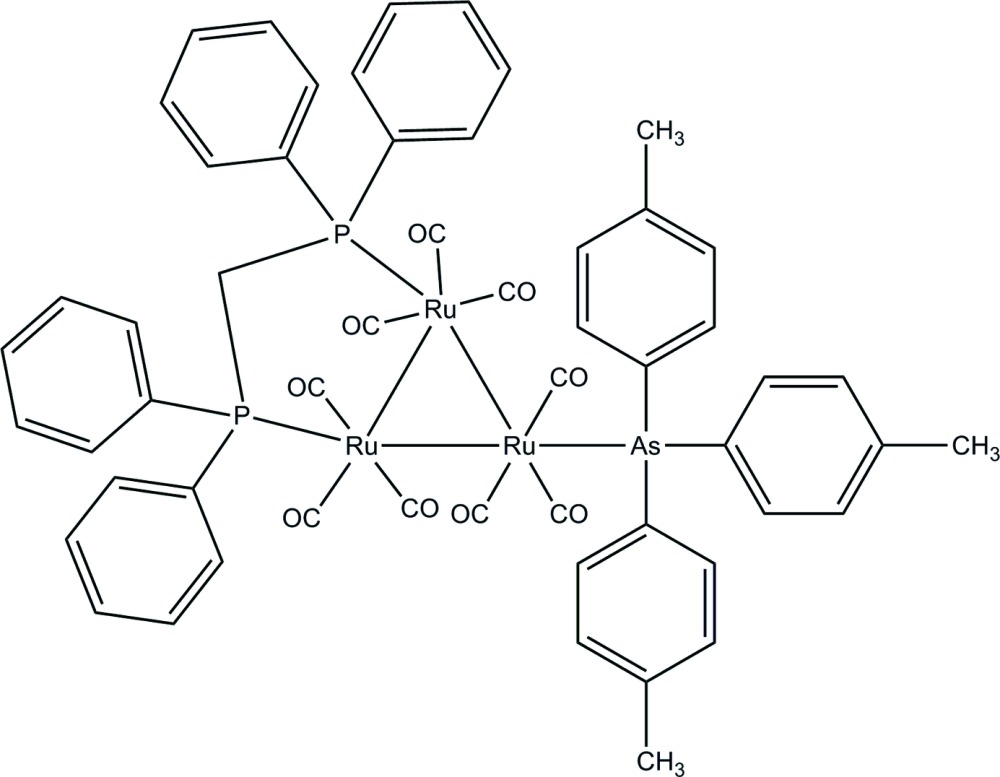


## Experimental

### 

#### Crystal data

[Ru_3_(C_21_H_21_As)(C_25_H_22_P_2_)(CO)_9_]
*M* *_r_* = 1287.96
Triclinic, 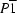

*a* = 11.1241 (1) Å
*b* = 12.1660 (2) Å
*c* = 20.0895 (3) Å
α = 91.406 (1)°
β = 101.116 (1)°
γ = 102.473 (1)°
*V* = 2598.63 (6) Å^3^
*Z* = 2
Mo *K*α radiation
μ = 1.61 mm^−1^
*T* = 100 K
0.29 × 0.22 × 0.06 mm

#### Data collection

Bruker SMART APEXII CCD area-detector diffractometer
Absorption correction: multi-scan (**SADABS**; Bruker, 2005 ▶) *T*_min_ = 0.653, *T*_max_ = 0.907
104298 measured reflections
24677 independent reflections
19683 reflections with *I* > 2σ(*I*)
*R*_int_ = 0.035

#### Refinement

*R*[*F* ^2^ > 2σ(*F* ^2^)] = 0.037
*wR*(*F* ^2^) = 0.084
*S* = 1.02
24677 reflections
634 parameters
H-atom parameters constrained
Δρ_max_ = 3.41 e Å^−3^
Δρ_min_ = −1.34 e Å^−3^

### 

Data collection: *APEX2* (Bruker, 2005 ▶); cell refinement: *SAINT* (Bruker, 2005 ▶); data reduction: *SAINT*; program(s) used to solve structure: *SHELXTL* (Sheldrick, 2008 ▶); program(s) used to refine structure: *SHELXTL*; molecular graphics: *SHELXTL*; software used to prepare material for publication: *SHELXTL* and *PLATON* (Spek, 2009 ▶).

## Supplementary Material

Crystal structure: contains datablocks global, I. DOI: 10.1107/S1600536809046704/sj2670sup1.cif

Structure factors: contains datablocks I. DOI: 10.1107/S1600536809046704/sj2670Isup2.hkl

Additional supplementary materials:  crystallographic information; 3D view; checkCIF report

## Figures and Tables

**Table 1:** Hydrogen-bond geometry (Å, °)

*D*—H⋯*A*	*D*—H	H⋯*A*	*D*⋯*A*	*D*—H⋯*A*
C16—H16*A*⋯O3^i^	0.93	2.49	3.377 (3)	159
C3—H3*A*⋯*Cg*1^ii^	0.93	2.93	3.708 (3)	142
C22—H22*A*⋯*Cg*2^iii^	0.93	2.78	3.453 (2)	130
C53—H53*B*⋯*Cg*3^iv^	0.96	2.94	3.801 (3)	150

Symmetry codes: (i) 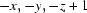; (ii) 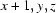; (iii) 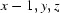; (iv) 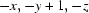. *Cg*1, *Cg*2 and *Cg*3 are centroids of the C20–C25 phenyl ring and C26–C32 and C38–C43 benzene rings, respectively.

**Fig. 1.**
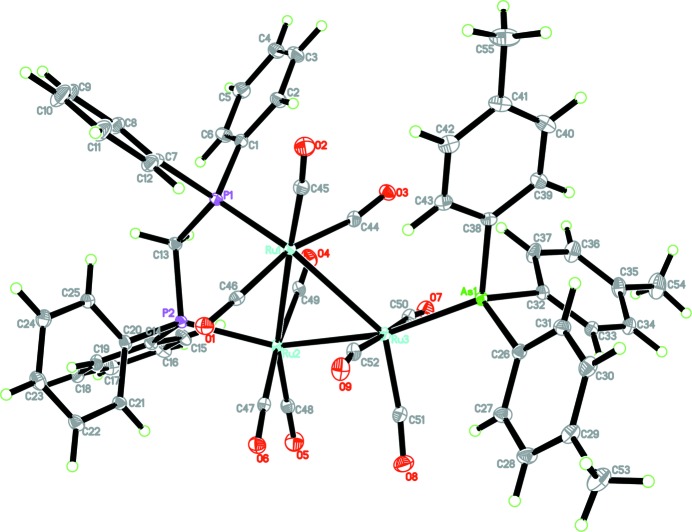
The molecular structure of the title compound with 30% probability ellipsoids for non-H atoms.

**Fig. 2.**
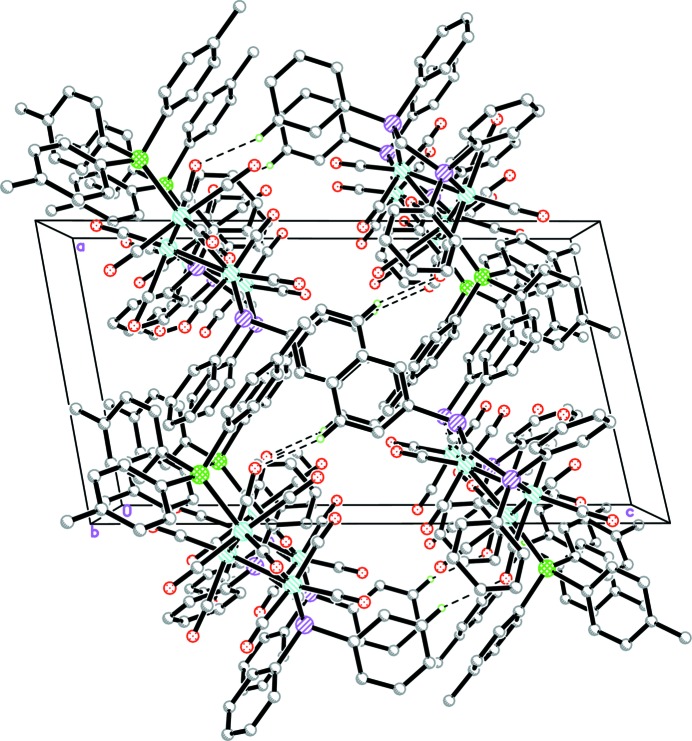
The crystal packing of the title compound, viewed down the b axis, showing the molecular dimers stacked down b axis. Hydrogen atoms that not involved in the hydrogen-bonding (dashed lines) have been omitted for clarity.

## supplementary crystallographic information

### Comment

Tri-angulotriruthenium clusters are known for their interesting structural variations and related catalytic activity. A large number of substituted derivatives, Ru_3_(CO)_12-n_*L*_n_ (*L*=15 group ligand) have been reported (Bruce *et al.*, 1988*a*,b; Bruce *et al.*, 1985). In continuation of our interest in the substituted clusters (Shawkataly *et al.*, 1998, 2009*a*) we report here the synthesis and structure of Ru_3_(CO)_9_(µ-Ph_2_PCH_2_PPh_2_)(As(4-CH_3_C_6_H_4_)_3_).

The bond lengths and angles of title compound (Fig. 1) are comparable to those in related structures (Shawkataly *et al.*, 2006, 2009*a*,b,c). The bis(diphenylphosphino)methane ligand bridges the Ru1—Ru2 bond and the monodentate arsine ligand bonds to the Ru3 atom. Both the phosphine and arsine ligands are equatorial with respect to the Ru_3_ triangle. Additionally, each Ru atom carries one equatorial and two axial terminal carbonyl ligands. The three arsine substituted phenyl rings make dihedral angles (C26–C31/C32–C37, C26–C31/C38–C43 and C32–C37/C38–C43) of 87.36 (10), 81.96 (10) and 73.37 (11)° with each other respectively. The dihedral angles between the two phenyl rings (C1–C6/C7–C12 and C14–C19/C20–C25) are 88.08 (12) and 80.15 (10)° for the two diphenylphosphino groups respectively.

In the crystal packing (Fig. 2), the molecules are linked together as dimers *via* intermolecular C—H···O hydrogen bonds. These dimers are stacked down *b* axis. Intermolecular C—H···π (Table 1) and π–π interactions further stabilize the crystal structure with *Cg*4···*Cg*4 = 3.6383 (13) Å, -1 - *x*, -*y*, 1 - *z* (*Cg*4 is centroid of phenyl ring C14–C19).

### Experimental

Reactions were conducted under an atmosphere of nitrogen using standard Schlenk techniques and hexane-dried over sodium metal. Tris(4-methylphenyl)arsine (Maybridge) is used as received and bis(diphenylphosphino)methane (Bruce *et al.*, 1983) was prepared by a reported procedure. Ru_3_(CO)_10_(µ-Ph_2_PCH_2_PPh_2_) (102.4 mg, 0.1 mmol) and tris(*p*-tolyl)arsine (34.83 mg, 0.1 mmol) were refluxed for 15 minutes in hexane (25 ml) under a current of nitrogen. The reaction mixture turned intense red. The solvent was removed under vacuum. The reaction mixture was separated by TLC (dichloromethane:hexane, 30:70). Two bands appeared. The major band (red) *R*_f_ = 0.56 yielded the title compound which was crystallized from CH_2_Cl_2_ - CH_3_OH, yield = 60 mg, 43.73%, m.p. 197–200 °C. IR(cyclohexane).ν(CO) 2082 s, 2000 s, 1981 s, cm^-1^. ^1^H NMR (CDCl_3_), δ 7.144–7.499 (32*H*, Ph), δ 4.185–4.336 (m, 2H, –CH_2_–), δ 2.365 (s, 9H, 3CH_3_).

### Refinement

All hydrogen atoms were positioned geometrically and refined using a riding model with C—H = 0.93–0.96 Å and *U*_iso_(H) = 1.2 or 1.5 *U*_eq_(C). A rotating group model was used for the methyl groups.

### Crystal data

**Table:**

[Ru_3_(C_21_H_21_As)(C_25_H_22_P_2_)(CO)_9_]	*Z* = 2
*M**_r_* = 1287.96	*F*(000) = 1280
Triclinic, *P*1	*D*_x_ = 1.646 Mg m^−^^3^
Hall symbol: -P 1	Mo *K*α radiation, λ = 0.71073 Å
*a* = 11.1241 (1) Å	Cell parameters from 9275 reflections
*b* = 12.1660 (2) Å	θ = 2.4–36.0°
*c* = 20.0895 (3) Å	µ = 1.61 mm^−^^1^
α = 91.406 (1)°	*T* = 100 K
β = 101.116 (1)°	Plate, red
γ = 102.473 (1)°	0.29 × 0.22 × 0.06 mm
*V* = 2598.63 (6) Å^3^	

### Data collection

**Table:**

Bruker SMART APEXII CCD area-detector diffractometer	24677 independent reflections
Radiation source: fine-focus sealed tube	19683 reflections with *I* > 2σ(*I*)
graphite	*R*_int_ = 0.035
φ and ω scans	θ_max_ = 36.2°, θ_min_ = 1.9°
Absorption correction: multi-scan (*SADABS*; Bruker, 2005)	*h* = −18→18
*T*_min_ = 0.653, *T*_max_ = 0.907	*k* = −20→19
104298 measured reflections	*l* = −33→32

### Refinement

**Table:**

Refinement on *F*^2^	Primary atom site location: structure-invariant direct methods
Least-squares matrix: full	Secondary atom site location: difference Fourier map
*R*[*F*^2^ > 2σ(*F*^2^)] = 0.037	Hydrogen site location: inferred from neighbouring sites
*wR*(*F*^2^) = 0.084	H-atom parameters constrained
*S* = 1.02	*w* = 1/[σ^2^(*F*_o_^2^) + (0.0322*P*)^2^ + 3.0613*P*] where *P* = (*F*_o_^2^ + 2*F*_c_^2^)/3
24677 reflections	(Δ/σ)_max_ = 0.001
634 parameters	Δρ_max_ = 3.41 e Å^−^^3^
0 restraints	Δρ_min_ = −1.34 e Å^−^^3^

### Special details

**Table:**

Experimental. The crystal was placed in the cold stream of an Oxford Cyrosystems Cobra open-flow nitrogen cryostat (Cosier & Glazer, 1986) operating at 100.0 (1) K. IR spectra was recorded with a Matson 1000 FTIR spectrometer in a NaCl solution cell (0.1 mm). NMR spectra recorded in CDCl_3_ with a Bruker 400 MHz s pectrometer.
Geometry. All e.s.d.'s (except the e.s.d. in the dihedral angle between two l.s. planes) are estimated using the full covariance matrix. The cell e.s.d.'s are taken into account individually in the estimation of e.s.d.'s in distances, angles and torsion angles; correlations between e.s.d.'s in cell parameters are only used when they are defined by crystal symmetry. An approximate (isotropic) treatment of cell e.s.d.'s is used for estimating e.s.d.'s involving l.s. planes.
Refinement. Refinement of *F*^2^ against ALL reflections. The weighted *R*-factor *wR* and goodness of fit *S* are based on *F*^2^, conventional *R*-factors *R* are based on *F*, with *F* set to zero for negative *F*^2^. The threshold expression of *F*^2^ > σ(*F*^2^) is used only for calculating *R*-factors(gt) *etc*. and is not relevant to the choice of reflections for refinement. *R*-factors based on *F*^2^ are statistically about twice as large as those based on *F*, and *R*- factors based on ALL data will be even larger.

### Fractional atomic coordinates and isotropic or equivalent isotropic displacement parameters (Å^2^)

**Table:**

	*x*	*y*	*z*	*U*_iso_*/*U*_eq_	
Ru1	−0.092501 (13)	0.023179 (12)	0.225967 (7)	0.01420 (3)	
Ru2	−0.184055 (13)	0.120663 (12)	0.329974 (7)	0.01335 (3)	
Ru3	−0.006333 (14)	0.260591 (12)	0.267982 (7)	0.01472 (3)	
As1	0.174870 (18)	0.346910 (16)	0.217792 (10)	0.01513 (4)	
P1	−0.14945 (4)	−0.15583 (4)	0.26340 (2)	0.01517 (8)	
P2	−0.32726 (4)	−0.04886 (4)	0.33226 (2)	0.01405 (8)	
O1	−0.35304 (14)	0.02944 (13)	0.14079 (7)	0.0228 (3)	
O2	−0.01281 (17)	−0.06563 (15)	0.10257 (8)	0.0307 (4)	
O3	0.18160 (14)	0.06485 (14)	0.30313 (8)	0.0233 (3)	
O4	0.01850 (14)	0.02577 (14)	0.42259 (8)	0.0251 (3)	
O5	−0.22301 (17)	0.26804 (15)	0.44509 (8)	0.0322 (4)	
O6	−0.36138 (14)	0.23945 (13)	0.23654 (8)	0.0244 (3)	
O7	0.16295 (16)	0.27361 (15)	0.41018 (8)	0.0313 (4)	
O8	−0.09867 (17)	0.45777 (14)	0.31728 (9)	0.0318 (4)	
O9	−0.17955 (16)	0.26067 (15)	0.12914 (8)	0.0285 (3)	
C1	−0.01915 (18)	−0.21580 (16)	0.30540 (10)	0.0184 (3)	
C2	0.0805 (2)	−0.21172 (19)	0.27234 (12)	0.0241 (4)	
H2A	0.0810	−0.1756	0.2321	0.029*	
C3	0.1795 (2)	−0.2612 (2)	0.29890 (13)	0.0279 (4)	
H3A	0.2453	−0.2584	0.2762	0.033*	
C4	0.1805 (2)	−0.3145 (2)	0.35887 (12)	0.0277 (4)	
H4A	0.2462	−0.3484	0.3761	0.033*	
C5	0.0834 (2)	−0.3173 (2)	0.39312 (12)	0.0272 (4)	
H5A	0.0844	−0.3522	0.4338	0.033*	
C6	−0.0160 (2)	−0.26779 (19)	0.36655 (11)	0.0239 (4)	
H6A	−0.0808	−0.2695	0.3899	0.029*	
C7	−0.23405 (18)	−0.27244 (16)	0.19979 (10)	0.0189 (3)	
C8	−0.2579 (2)	−0.38311 (18)	0.22004 (12)	0.0256 (4)	
H8A	−0.2311	−0.3969	0.2652	0.031*	
C9	−0.3213 (3)	−0.4723 (2)	0.17321 (14)	0.0357 (5)	
H9A	−0.3373	−0.5455	0.1871	0.043*	
C10	−0.3608 (3)	−0.4528 (2)	0.10588 (15)	0.0389 (6)	
H10A	−0.4030	−0.5128	0.0746	0.047*	
C11	−0.3375 (2)	−0.3440 (2)	0.08499 (13)	0.0323 (5)	
H11A	−0.3639	−0.3310	0.0397	0.039*	
C12	−0.2743 (2)	−0.25394 (18)	0.13192 (11)	0.0236 (4)	
H12A	−0.2590	−0.1809	0.1177	0.028*	
C13	−0.25005 (17)	−0.16738 (16)	0.32744 (10)	0.0169 (3)	
H13A	−0.3142	−0.2368	0.3169	0.020*	
H13B	−0.1990	−0.1724	0.3717	0.020*	
C14	−0.38444 (17)	−0.07840 (16)	0.41085 (9)	0.0165 (3)	
C15	−0.29807 (19)	−0.0528 (2)	0.47252 (10)	0.0236 (4)	
H15A	−0.2149	−0.0177	0.4728	0.028*	
C16	−0.3348 (2)	−0.0791 (2)	0.53344 (11)	0.0268 (4)	
H16A	−0.2764	−0.0623	0.5742	0.032*	
C17	−0.4591 (2)	−0.13053 (19)	0.53333 (11)	0.0248 (4)	
H17A	−0.4841	−0.1481	0.5741	0.030*	
C18	−0.5454 (2)	−0.15564 (19)	0.47262 (11)	0.0247 (4)	
H18A	−0.6286	−0.1899	0.4726	0.030*	
C19	−0.50858 (19)	−0.12991 (17)	0.41136 (10)	0.0206 (4)	
H19A	−0.5672	−0.1472	0.3707	0.025*	
C20	−0.46895 (16)	−0.08207 (16)	0.26597 (9)	0.0155 (3)	
C21	−0.54723 (17)	−0.00499 (16)	0.25740 (9)	0.0169 (3)	
H21A	−0.5280	0.0595	0.2867	0.020*	
C22	−0.65329 (18)	−0.02445 (18)	0.20536 (10)	0.0206 (4)	
H22A	−0.7048	0.0269	0.2000	0.025*	
C23	−0.68229 (19)	−0.1205 (2)	0.16137 (11)	0.0242 (4)	
H23A	−0.7524	−0.1328	0.1261	0.029*	
C24	−0.6067 (2)	−0.19810 (19)	0.17000 (11)	0.0241 (4)	
H24A	−0.6269	−0.2629	0.1409	0.029*	
C25	−0.50038 (18)	−0.17913 (17)	0.22222 (10)	0.0196 (3)	
H25A	−0.4502	−0.2316	0.2279	0.024*	
C26	0.14521 (18)	0.46556 (16)	0.15728 (10)	0.0175 (3)	
C27	0.0521 (2)	0.52311 (18)	0.16335 (11)	0.0230 (4)	
H27A	0.0087	0.5090	0.1985	0.028*	
C28	0.0236 (2)	0.60180 (19)	0.11705 (11)	0.0257 (4)	
H28A	−0.0388	0.6397	0.1218	0.031*	
C29	0.0867 (2)	0.62468 (17)	0.06387 (10)	0.0224 (4)	
C30	0.1811 (2)	0.56661 (19)	0.05844 (11)	0.0253 (4)	
H30A	0.2248	0.5806	0.0233	0.030*	
C31	0.2101 (2)	0.48899 (18)	0.10459 (11)	0.0221 (4)	
H31A	0.2735	0.4520	0.1004	0.027*	
C32	0.32589 (17)	0.41783 (16)	0.28350 (10)	0.0178 (3)	
C33	0.37183 (18)	0.53379 (17)	0.28934 (10)	0.0201 (3)	
H33A	0.3344	0.5787	0.2588	0.024*	
C34	0.4737 (2)	0.58329 (18)	0.34080 (12)	0.0249 (4)	
H34A	0.5024	0.6613	0.3447	0.030*	
C35	0.5331 (2)	0.5188 (2)	0.38627 (12)	0.0282 (4)	
C36	0.4884 (2)	0.4020 (2)	0.37915 (13)	0.0323 (5)	
H36A	0.5283	0.3571	0.4087	0.039*	
C37	0.3853 (2)	0.35127 (19)	0.32867 (12)	0.0271 (4)	
H37A	0.3560	0.2733	0.3250	0.032*	
C38	0.24235 (18)	0.25781 (16)	0.15858 (10)	0.0180 (3)	
C39	0.37055 (19)	0.27305 (19)	0.16030 (11)	0.0228 (4)	
H39A	0.4281	0.3234	0.1930	0.027*	
C40	0.4128 (2)	0.2133 (2)	0.11325 (11)	0.0270 (4)	
H40A	0.4987	0.2243	0.1147	0.032*	
C41	0.3282 (2)	0.1367 (2)	0.06356 (11)	0.0259 (4)	
C42	0.2006 (2)	0.12344 (19)	0.06198 (10)	0.0248 (4)	
H42A	0.1430	0.0737	0.0290	0.030*	
C43	0.1574 (2)	0.18324 (18)	0.10882 (10)	0.0208 (4)	
H43A	0.0715	0.1733	0.1068	0.025*	
C44	0.07788 (19)	0.05990 (17)	0.27751 (10)	0.0193 (3)	
C45	−0.03974 (19)	−0.02775 (18)	0.14906 (10)	0.0205 (3)	
C46	−0.25780 (19)	0.02840 (16)	0.17457 (10)	0.0180 (3)	
C47	−0.29468 (18)	0.19138 (16)	0.26811 (9)	0.0173 (3)	
C48	−0.20782 (18)	0.21153 (18)	0.40222 (10)	0.0199 (3)	
C49	−0.05406 (18)	0.06182 (17)	0.38594 (10)	0.0186 (3)	
C50	0.0998 (2)	0.26239 (17)	0.35718 (10)	0.0210 (4)	
C51	−0.05753 (19)	0.38722 (17)	0.29861 (10)	0.0208 (4)	
C52	−0.11750 (19)	0.25061 (18)	0.18030 (10)	0.0208 (4)	
C53	0.0546 (3)	0.7065 (2)	0.01282 (11)	0.0315 (5)	
H53A	−0.0010	0.7474	0.0280	0.047*	
H53B	0.0140	0.6662	−0.0301	0.047*	
H53C	0.1301	0.7584	0.0078	0.047*	
C54	0.6437 (3)	0.5730 (3)	0.44273 (15)	0.0442 (7)	
H54A	0.6470	0.6523	0.4484	0.066*	
H54B	0.7203	0.5625	0.4310	0.066*	
H54C	0.6336	0.5386	0.4844	0.066*	
C55	0.3739 (3)	0.0679 (3)	0.01549 (12)	0.0391 (6)	
H55A	0.3033	0.0187	−0.0137	0.059*	
H55B	0.4264	0.0237	0.0408	0.059*	
H55C	0.4215	0.1170	−0.0115	0.059*	

### Atomic displacement parameters (Å^2^)

**Table:**

	*U*^11^	*U*^22^	*U*^33^	*U*^12^	*U*^13^	*U*^23^
Ru1	0.01525 (6)	0.01428 (6)	0.01282 (6)	0.00304 (5)	0.00261 (4)	0.00064 (5)
Ru2	0.01196 (6)	0.01505 (6)	0.01221 (5)	0.00194 (5)	0.00173 (4)	0.00064 (4)
Ru3	0.01490 (6)	0.01372 (6)	0.01460 (6)	0.00119 (5)	0.00306 (5)	0.00016 (5)
As1	0.01466 (8)	0.01405 (8)	0.01656 (8)	0.00346 (6)	0.00254 (6)	0.00169 (6)
P1	0.01332 (19)	0.0149 (2)	0.0173 (2)	0.00427 (16)	0.00200 (15)	0.00145 (16)
P2	0.01206 (18)	0.0156 (2)	0.01410 (18)	0.00291 (15)	0.00175 (14)	0.00185 (15)
O1	0.0227 (7)	0.0262 (7)	0.0189 (6)	0.0075 (6)	0.0004 (5)	−0.0001 (5)
O2	0.0337 (9)	0.0331 (9)	0.0253 (8)	0.0052 (7)	0.0099 (7)	−0.0068 (7)
O3	0.0183 (6)	0.0279 (8)	0.0228 (7)	0.0055 (6)	0.0020 (5)	−0.0028 (6)
O4	0.0177 (6)	0.0336 (8)	0.0233 (7)	0.0063 (6)	0.0012 (5)	0.0080 (6)
O5	0.0380 (9)	0.0348 (9)	0.0240 (7)	0.0046 (7)	0.0115 (7)	−0.0070 (7)
O6	0.0212 (7)	0.0253 (7)	0.0269 (7)	0.0076 (6)	0.0016 (6)	0.0072 (6)
O7	0.0311 (8)	0.0325 (9)	0.0236 (7)	0.0011 (7)	−0.0040 (6)	0.0019 (6)
O8	0.0375 (9)	0.0248 (8)	0.0358 (9)	0.0107 (7)	0.0098 (7)	−0.0001 (7)
O9	0.0288 (8)	0.0299 (8)	0.0224 (7)	0.0011 (7)	0.0003 (6)	0.0072 (6)
C1	0.0150 (7)	0.0170 (8)	0.0227 (8)	0.0049 (6)	0.0013 (6)	0.0013 (7)
C2	0.0207 (9)	0.0261 (10)	0.0280 (10)	0.0087 (8)	0.0067 (7)	0.0055 (8)
C3	0.0193 (9)	0.0308 (11)	0.0361 (12)	0.0104 (8)	0.0065 (8)	0.0042 (9)
C4	0.0185 (9)	0.0279 (11)	0.0356 (11)	0.0094 (8)	−0.0014 (8)	0.0015 (9)
C5	0.0268 (10)	0.0284 (11)	0.0265 (10)	0.0115 (9)	−0.0008 (8)	0.0041 (8)
C6	0.0209 (9)	0.0281 (10)	0.0238 (9)	0.0106 (8)	0.0013 (7)	0.0040 (8)
C7	0.0163 (8)	0.0167 (8)	0.0238 (9)	0.0058 (6)	0.0021 (6)	−0.0015 (7)
C8	0.0274 (10)	0.0167 (9)	0.0314 (11)	0.0045 (8)	0.0031 (8)	0.0017 (8)
C9	0.0410 (14)	0.0175 (10)	0.0435 (14)	0.0022 (9)	0.0018 (11)	−0.0011 (9)
C10	0.0448 (15)	0.0226 (11)	0.0407 (14)	0.0023 (10)	−0.0047 (11)	−0.0102 (10)
C11	0.0375 (13)	0.0260 (11)	0.0280 (11)	0.0063 (10)	−0.0043 (9)	−0.0055 (9)
C12	0.0246 (9)	0.0185 (9)	0.0253 (9)	0.0045 (7)	0.0002 (7)	−0.0025 (7)
C13	0.0152 (7)	0.0174 (8)	0.0191 (8)	0.0054 (6)	0.0033 (6)	0.0031 (6)
C14	0.0158 (7)	0.0179 (8)	0.0172 (7)	0.0055 (6)	0.0047 (6)	0.0046 (6)
C15	0.0167 (8)	0.0359 (11)	0.0191 (8)	0.0084 (8)	0.0026 (6)	0.0062 (8)
C16	0.0265 (10)	0.0383 (12)	0.0176 (8)	0.0112 (9)	0.0046 (7)	0.0083 (8)
C17	0.0326 (11)	0.0247 (10)	0.0215 (9)	0.0088 (8)	0.0124 (8)	0.0090 (8)
C18	0.0262 (10)	0.0230 (9)	0.0256 (9)	0.0003 (8)	0.0122 (8)	0.0035 (8)
C19	0.0196 (8)	0.0199 (9)	0.0212 (8)	0.0008 (7)	0.0057 (7)	0.0010 (7)
C20	0.0131 (7)	0.0167 (8)	0.0157 (7)	0.0022 (6)	0.0017 (6)	0.0025 (6)
C21	0.0150 (7)	0.0182 (8)	0.0176 (7)	0.0048 (6)	0.0020 (6)	0.0027 (6)
C22	0.0164 (8)	0.0252 (9)	0.0208 (8)	0.0071 (7)	0.0021 (6)	0.0065 (7)
C23	0.0173 (8)	0.0315 (11)	0.0210 (9)	0.0045 (8)	−0.0022 (7)	0.0023 (8)
C24	0.0210 (9)	0.0243 (10)	0.0229 (9)	0.0023 (8)	−0.0016 (7)	−0.0052 (7)
C25	0.0166 (8)	0.0192 (8)	0.0219 (8)	0.0035 (7)	0.0019 (6)	−0.0010 (7)
C26	0.0174 (8)	0.0158 (8)	0.0183 (8)	0.0033 (6)	0.0015 (6)	0.0024 (6)
C27	0.0265 (10)	0.0234 (9)	0.0230 (9)	0.0107 (8)	0.0077 (7)	0.0075 (7)
C28	0.0289 (10)	0.0249 (10)	0.0268 (10)	0.0127 (8)	0.0056 (8)	0.0081 (8)
C29	0.0287 (10)	0.0187 (9)	0.0165 (8)	0.0042 (7)	−0.0021 (7)	0.0024 (7)
C30	0.0327 (11)	0.0222 (9)	0.0210 (9)	0.0036 (8)	0.0076 (8)	0.0057 (7)
C31	0.0238 (9)	0.0211 (9)	0.0240 (9)	0.0071 (7)	0.0084 (7)	0.0042 (7)
C32	0.0156 (7)	0.0184 (8)	0.0183 (8)	0.0019 (6)	0.0026 (6)	0.0011 (6)
C33	0.0185 (8)	0.0170 (8)	0.0243 (9)	0.0033 (7)	0.0038 (7)	0.0017 (7)
C34	0.0213 (9)	0.0192 (9)	0.0306 (10)	−0.0015 (7)	0.0040 (8)	−0.0017 (8)
C35	0.0203 (9)	0.0327 (11)	0.0252 (10)	−0.0032 (8)	−0.0007 (7)	0.0011 (8)
C36	0.0276 (11)	0.0299 (11)	0.0317 (11)	0.0007 (9)	−0.0069 (9)	0.0093 (9)
C37	0.0252 (10)	0.0200 (9)	0.0295 (10)	0.0000 (8)	−0.0050 (8)	0.0072 (8)
C38	0.0197 (8)	0.0170 (8)	0.0187 (8)	0.0073 (7)	0.0038 (6)	0.0019 (6)
C39	0.0189 (8)	0.0258 (10)	0.0250 (9)	0.0078 (7)	0.0044 (7)	0.0018 (8)
C40	0.0238 (10)	0.0378 (12)	0.0233 (9)	0.0136 (9)	0.0066 (8)	0.0047 (9)
C41	0.0334 (11)	0.0327 (11)	0.0178 (8)	0.0189 (9)	0.0066 (8)	0.0049 (8)
C42	0.0313 (11)	0.0266 (10)	0.0170 (8)	0.0097 (8)	0.0023 (7)	0.0020 (7)
C43	0.0230 (9)	0.0216 (9)	0.0183 (8)	0.0079 (7)	0.0023 (7)	0.0017 (7)
C44	0.0207 (8)	0.0199 (8)	0.0160 (7)	0.0011 (7)	0.0046 (6)	−0.0010 (6)
C45	0.0206 (8)	0.0213 (9)	0.0186 (8)	0.0043 (7)	0.0026 (6)	−0.0001 (7)
C46	0.0219 (8)	0.0158 (8)	0.0169 (8)	0.0048 (7)	0.0047 (6)	0.0007 (6)
C47	0.0168 (8)	0.0158 (8)	0.0176 (8)	0.0009 (6)	0.0029 (6)	0.0004 (6)
C48	0.0187 (8)	0.0226 (9)	0.0177 (8)	0.0028 (7)	0.0042 (6)	0.0013 (7)
C49	0.0150 (7)	0.0216 (9)	0.0186 (8)	0.0022 (7)	0.0045 (6)	0.0021 (7)
C50	0.0224 (9)	0.0174 (8)	0.0218 (8)	0.0017 (7)	0.0043 (7)	0.0017 (7)
C51	0.0220 (9)	0.0185 (8)	0.0203 (8)	0.0012 (7)	0.0042 (7)	0.0003 (7)
C52	0.0189 (8)	0.0214 (9)	0.0208 (8)	0.0009 (7)	0.0051 (7)	0.0026 (7)
C53	0.0474 (14)	0.0250 (10)	0.0195 (9)	0.0073 (10)	0.0006 (9)	0.0063 (8)
C54	0.0340 (13)	0.0455 (16)	0.0374 (14)	−0.0084 (12)	−0.0116 (11)	0.0021 (12)
C55	0.0576 (17)	0.0488 (16)	0.0203 (10)	0.0313 (14)	0.0094 (10)	0.0017 (10)

### Geometric parameters (Å, °)

**Table:**

Ru1—C45	1.891 (2)	C16—C17	1.388 (3)
Ru1—C44	1.928 (2)	C16—H16A	0.9300
Ru1—C46	1.942 (2)	C17—C18	1.381 (3)
Ru1—P1	2.3190 (5)	C17—H17A	0.9300
Ru1—Ru2	2.8338 (2)	C18—C19	1.392 (3)
Ru1—Ru3	2.8890 (2)	C18—H18A	0.9300
Ru2—C48	1.896 (2)	C19—H19A	0.9300
Ru2—C47	1.9286 (19)	C20—C25	1.393 (3)
Ru2—C49	1.931 (2)	C20—C21	1.404 (3)
Ru2—P2	2.3253 (5)	C21—C22	1.390 (3)
Ru2—Ru3	2.8208 (2)	C21—H21A	0.9300
Ru3—C51	1.883 (2)	C22—C23	1.388 (3)
Ru3—C52	1.932 (2)	C22—H22A	0.9300
Ru3—C50	1.943 (2)	C23—C24	1.387 (3)
Ru3—As1	2.4706 (2)	C23—H23A	0.9300
As1—C32	1.9422 (19)	C24—C25	1.394 (3)
As1—C38	1.9458 (19)	C24—H24A	0.9300
As1—C26	1.9513 (19)	C25—H25A	0.9300
P1—C1	1.8332 (19)	C26—C27	1.391 (3)
P1—C7	1.844 (2)	C26—C31	1.394 (3)
P1—C13	1.8503 (19)	C27—C28	1.394 (3)
P2—C20	1.8193 (18)	C27—H27A	0.9300
P2—C14	1.8283 (19)	C28—C29	1.390 (3)
P2—C13	1.8402 (19)	C28—H28A	0.9300
O1—C46	1.145 (2)	C29—C30	1.406 (3)
O2—C45	1.149 (2)	C29—C53	1.494 (3)
O3—C44	1.157 (2)	C30—C31	1.384 (3)
O4—C49	1.151 (2)	C30—H30A	0.9300
O5—C48	1.149 (3)	C31—H31A	0.9300
O6—C47	1.148 (2)	C32—C33	1.387 (3)
O7—C50	1.144 (3)	C32—C37	1.397 (3)
O8—C51	1.143 (3)	C33—C34	1.392 (3)
O9—C52	1.147 (3)	C33—H33A	0.9300
C1—C2	1.390 (3)	C34—C35	1.383 (3)
C1—C6	1.393 (3)	C34—H34A	0.9300
C2—C3	1.391 (3)	C35—C36	1.394 (3)
C2—H2A	0.9300	C35—C54	1.520 (3)
C3—C4	1.381 (3)	C36—C37	1.393 (3)
C3—H3A	0.9300	C36—H36A	0.9300
C4—C5	1.383 (3)	C37—H37A	0.9300
C4—H4A	0.9300	C38—C39	1.391 (3)
C5—C6	1.395 (3)	C38—C43	1.392 (3)
C5—H5A	0.9300	C39—C40	1.388 (3)
C6—H6A	0.9300	C39—H39A	0.9300
C7—C12	1.391 (3)	C40—C41	1.401 (3)
C7—C8	1.401 (3)	C40—H40A	0.9300
C8—C9	1.387 (3)	C41—C42	1.387 (3)
C8—H8A	0.9300	C41—C55	1.492 (3)
C9—C10	1.382 (4)	C42—C43	1.392 (3)
C9—H9A	0.9300	C42—H42A	0.9300
C10—C11	1.384 (4)	C43—H43A	0.9300
C10—H10A	0.9300	C53—H53A	0.9600
C11—C12	1.394 (3)	C53—H53B	0.9600
C11—H11A	0.9300	C53—H53C	0.9600
C12—H12A	0.9300	C54—H54A	0.9600
C13—H13A	0.9700	C54—H54B	0.9600
C13—H13B	0.9700	C54—H54C	0.9600
C14—C19	1.390 (3)	C55—H55A	0.9600
C14—C15	1.396 (3)	C55—H55B	0.9600
C15—C16	1.386 (3)	C55—H55C	0.9600
C15—H15A	0.9300		
			
C45—Ru1—C44	91.37 (8)	C15—C16—C17	119.8 (2)
C45—Ru1—C46	93.46 (8)	C15—C16—H16A	120.1
C44—Ru1—C46	165.13 (8)	C17—C16—H16A	120.1
C45—Ru1—P1	94.42 (6)	C18—C17—C16	119.91 (19)
C44—Ru1—P1	96.98 (6)	C18—C17—H17A	120.0
C46—Ru1—P1	96.66 (6)	C16—C17—H17A	120.0
C45—Ru1—Ru2	172.35 (6)	C17—C18—C19	120.4 (2)
C44—Ru1—Ru2	92.49 (6)	C17—C18—H18A	119.8
C46—Ru1—Ru2	81.22 (6)	C19—C18—H18A	119.8
P1—Ru1—Ru2	91.664 (13)	C14—C19—C18	120.20 (19)
C45—Ru1—Ru3	116.73 (6)	C14—C19—H19A	119.9
C44—Ru1—Ru3	67.59 (6)	C18—C19—H19A	119.9
C46—Ru1—Ru3	97.73 (6)	C25—C20—C21	118.95 (17)
P1—Ru1—Ru3	144.599 (14)	C25—C20—P2	122.50 (14)
Ru2—Ru1—Ru3	59.055 (5)	C21—C20—P2	118.52 (14)
C48—Ru2—C47	88.44 (8)	C22—C21—C20	120.43 (18)
C48—Ru2—C49	94.40 (8)	C22—C21—H21A	119.8
C47—Ru2—C49	171.67 (8)	C20—C21—H21A	119.8
C48—Ru2—P2	103.24 (6)	C23—C22—C21	120.03 (19)
C47—Ru2—P2	97.15 (6)	C23—C22—H22A	120.0
C49—Ru2—P2	89.84 (6)	C21—C22—H22A	120.0
C48—Ru2—Ru3	105.92 (6)	C24—C23—C22	120.04 (19)
C47—Ru2—Ru3	80.25 (6)	C24—C23—H23A	120.0
C49—Ru2—Ru3	91.43 (6)	C22—C23—H23A	120.0
P2—Ru2—Ru3	150.627 (13)	C23—C24—C25	120.16 (19)
C48—Ru2—Ru1	166.17 (6)	C23—C24—H24A	119.9
C47—Ru2—Ru1	94.46 (6)	C25—C24—H24A	119.9
C49—Ru2—Ru1	80.97 (6)	C20—C25—C24	120.38 (19)
P2—Ru2—Ru1	89.842 (13)	C20—C25—H25A	119.8
Ru3—Ru2—Ru1	61.448 (5)	C24—C25—H25A	119.8
C51—Ru3—C52	92.34 (9)	C27—C26—C31	118.77 (18)
C51—Ru3—C50	89.02 (9)	C27—C26—As1	120.03 (15)
C52—Ru3—C50	176.73 (8)	C31—C26—As1	121.06 (15)
C51—Ru3—As1	102.66 (6)	C26—C27—C28	120.5 (2)
C52—Ru3—As1	90.29 (6)	C26—C27—H27A	119.8
C50—Ru3—As1	92.32 (6)	C28—C27—H27A	119.8
C51—Ru3—Ru2	89.27 (6)	C29—C28—C27	121.2 (2)
C52—Ru3—Ru2	95.19 (6)	C29—C28—H28A	119.4
C50—Ru3—Ru2	81.84 (6)	C27—C28—H28A	119.4
As1—Ru3—Ru2	166.672 (9)	C28—C29—C30	117.92 (19)
C51—Ru3—Ru1	143.34 (6)	C28—C29—C53	121.7 (2)
C52—Ru3—Ru1	73.64 (6)	C30—C29—C53	120.4 (2)
C50—Ru3—Ru1	103.55 (6)	C31—C30—C29	120.9 (2)
As1—Ru3—Ru1	110.910 (8)	C31—C30—H30A	119.5
Ru2—Ru3—Ru1	59.496 (5)	C29—C30—H30A	119.5
C32—As1—C38	101.24 (8)	C30—C31—C26	120.7 (2)
C32—As1—C26	104.04 (8)	C30—C31—H31A	119.6
C38—As1—C26	98.60 (8)	C26—C31—H31A	119.6
C32—As1—Ru3	114.70 (6)	C33—C32—C37	119.16 (18)
C38—As1—Ru3	121.17 (6)	C33—C32—As1	121.50 (15)
C26—As1—Ru3	114.49 (6)	C37—C32—As1	119.23 (15)
C1—P1—C7	100.13 (9)	C32—C33—C34	120.27 (19)
C1—P1—C13	102.22 (9)	C32—C33—H33A	119.9
C7—P1—C13	102.95 (9)	C34—C33—H33A	119.9
C1—P1—Ru1	115.63 (6)	C35—C34—C33	121.3 (2)
C7—P1—Ru1	118.28 (7)	C35—C34—H34A	119.3
C13—P1—Ru1	115.20 (6)	C33—C34—H34A	119.3
C20—P2—C14	104.13 (8)	C34—C35—C36	118.2 (2)
C20—P2—C13	105.86 (9)	C34—C35—C54	121.3 (2)
C14—P2—C13	99.07 (9)	C36—C35—C54	120.6 (2)
C20—P2—Ru2	117.91 (6)	C37—C36—C35	121.2 (2)
C14—P2—Ru2	117.92 (6)	C37—C36—H36A	119.4
C13—P2—Ru2	109.76 (6)	C35—C36—H36A	119.4
C2—C1—C6	118.60 (18)	C36—C37—C32	119.8 (2)
C2—C1—P1	117.10 (15)	C36—C37—H37A	120.1
C6—C1—P1	124.27 (15)	C32—C37—H37A	120.1
C1—C2—C3	120.6 (2)	C39—C38—C43	119.29 (18)
C1—C2—H2A	119.7	C39—C38—As1	122.65 (15)
C3—C2—H2A	119.7	C43—C38—As1	117.81 (15)
C4—C3—C2	120.3 (2)	C40—C39—C38	120.1 (2)
C4—C3—H3A	119.8	C40—C39—H39A	119.9
C2—C3—H3A	119.8	C38—C39—H39A	119.9
C3—C4—C5	119.8 (2)	C39—C40—C41	121.1 (2)
C3—C4—H4A	120.1	C39—C40—H40A	119.4
C5—C4—H4A	120.1	C41—C40—H40A	119.4
C4—C5—C6	120.0 (2)	C42—C41—C40	118.1 (2)
C4—C5—H5A	120.0	C42—C41—C55	120.8 (2)
C6—C5—H5A	120.0	C40—C41—C55	121.0 (2)
C1—C6—C5	120.7 (2)	C41—C42—C43	121.2 (2)
C1—C6—H6A	119.7	C41—C42—H42A	119.4
C5—C6—H6A	119.7	C43—C42—H42A	119.4
C12—C7—C8	118.79 (19)	C38—C43—C42	120.17 (19)
C12—C7—P1	121.89 (15)	C38—C43—H43A	119.9
C8—C7—P1	119.32 (16)	C42—C43—H43A	119.9
C9—C8—C7	120.5 (2)	O3—C44—Ru1	168.27 (18)
C9—C8—H8A	119.8	O2—C45—Ru1	175.42 (19)
C7—C8—H8A	119.8	O1—C46—Ru1	175.83 (17)
C10—C9—C8	120.1 (2)	O6—C47—Ru2	173.52 (17)
C10—C9—H9A	119.9	O5—C48—Ru2	178.6 (2)
C8—C9—H9A	119.9	O4—C49—Ru2	175.36 (17)
C9—C10—C11	120.1 (2)	O7—C50—Ru3	173.88 (19)
C9—C10—H10A	119.9	O8—C51—Ru3	173.89 (19)
C11—C10—H10A	119.9	O9—C52—Ru3	170.53 (19)
C10—C11—C12	120.0 (2)	C29—C53—H53A	109.5
C10—C11—H11A	120.0	C29—C53—H53B	109.5
C12—C11—H11A	120.0	H53A—C53—H53B	109.5
C7—C12—C11	120.5 (2)	C29—C53—H53C	109.5
C7—C12—H12A	119.8	H53A—C53—H53C	109.5
C11—C12—H12A	119.8	H53B—C53—H53C	109.5
P2—C13—P1	114.18 (10)	C35—C54—H54A	109.5
P2—C13—H13A	108.7	C35—C54—H54B	109.5
P1—C13—H13A	108.7	H54A—C54—H54B	109.5
P2—C13—H13B	108.7	C35—C54—H54C	109.5
P1—C13—H13B	108.7	H54A—C54—H54C	109.5
H13A—C13—H13B	107.6	H54B—C54—H54C	109.5
C19—C14—C15	118.95 (18)	C41—C55—H55A	109.5
C19—C14—P2	122.70 (15)	C41—C55—H55B	109.5
C15—C14—P2	118.28 (14)	H55A—C55—H55B	109.5
C16—C15—C14	120.74 (19)	C41—C55—H55C	109.5
C16—C15—H15A	119.6	H55A—C55—H55C	109.5
C14—C15—H15A	119.6	H55B—C55—H55C	109.5
			
C44—Ru1—Ru2—C48	36.6 (3)	Ru1—P1—C1—C2	−49.09 (18)
C46—Ru1—Ru2—C48	−129.9 (3)	C7—P1—C1—C6	−98.64 (19)
P1—Ru1—Ru2—C48	133.7 (3)	C13—P1—C1—C6	7.1 (2)
Ru3—Ru1—Ru2—C48	−25.3 (3)	Ru1—P1—C1—C6	133.08 (17)
C44—Ru1—Ru2—C47	138.26 (8)	C6—C1—C2—C3	1.7 (3)
C46—Ru1—Ru2—C47	−28.19 (8)	P1—C1—C2—C3	−176.25 (18)
P1—Ru1—Ru2—C47	−124.67 (6)	C1—C2—C3—C4	−0.5 (4)
Ru3—Ru1—Ru2—C47	76.36 (5)	C2—C3—C4—C5	−0.9 (4)
C44—Ru1—Ru2—C49	−34.71 (8)	C3—C4—C5—C6	0.9 (4)
C46—Ru1—Ru2—C49	158.83 (8)	C2—C1—C6—C5	−1.7 (3)
P1—Ru1—Ru2—C49	62.35 (6)	P1—C1—C6—C5	176.15 (17)
Ru3—Ru1—Ru2—C49	−96.62 (6)	C4—C5—C6—C1	0.4 (3)
C44—Ru1—Ru2—P2	−124.58 (6)	C1—P1—C7—C12	−132.65 (18)
C46—Ru1—Ru2—P2	68.96 (6)	C13—P1—C7—C12	122.18 (18)
P1—Ru1—Ru2—P2	−27.517 (17)	Ru1—P1—C7—C12	−6.1 (2)
Ru3—Ru1—Ru2—P2	173.516 (13)	C1—P1—C7—C8	46.89 (18)
C44—Ru1—Ru2—Ru3	61.90 (6)	C13—P1—C7—C8	−58.28 (18)
C46—Ru1—Ru2—Ru3	−104.55 (6)	Ru1—P1—C7—C8	173.41 (14)
P1—Ru1—Ru2—Ru3	158.967 (13)	C12—C7—C8—C9	−0.4 (3)
C48—Ru2—Ru3—C51	−26.27 (9)	P1—C7—C8—C9	−179.98 (19)
C47—Ru2—Ru3—C51	59.27 (8)	C7—C8—C9—C10	0.5 (4)
C49—Ru2—Ru3—C51	−121.25 (9)	C8—C9—C10—C11	−0.2 (4)
P2—Ru2—Ru3—C51	146.53 (7)	C9—C10—C11—C12	−0.1 (4)
Ru1—Ru2—Ru3—C51	159.84 (6)	C8—C7—C12—C11	0.1 (3)
C48—Ru2—Ru3—C52	−118.55 (9)	P1—C7—C12—C11	179.64 (18)
C47—Ru2—Ru3—C52	−33.01 (9)	C10—C11—C12—C7	0.2 (4)
C49—Ru2—Ru3—C52	146.47 (9)	C20—P2—C13—P1	84.11 (11)
P2—Ru2—Ru3—C52	54.24 (7)	C14—P2—C13—P1	−168.29 (10)
Ru1—Ru2—Ru3—C52	67.55 (6)	Ru2—P2—C13—P1	−44.14 (11)
C48—Ru2—Ru3—C50	62.84 (9)	C1—P1—C13—P2	143.73 (10)
C47—Ru2—Ru3—C50	148.38 (9)	C7—P1—C13—P2	−112.71 (11)
C49—Ru2—Ru3—C50	−32.14 (9)	Ru1—P1—C13—P2	17.50 (12)
P2—Ru2—Ru3—C50	−124.36 (7)	C20—P2—C14—C19	7.93 (19)
Ru1—Ru2—Ru3—C50	−111.05 (6)	C13—P2—C14—C19	−101.07 (18)
C48—Ru2—Ru3—As1	127.52 (7)	Ru2—P2—C14—C19	140.75 (15)
C47—Ru2—Ru3—As1	−146.94 (7)	C20—P2—C14—C15	−175.16 (17)
C49—Ru2—Ru3—As1	32.54 (7)	C13—P2—C14—C15	75.84 (18)
P2—Ru2—Ru3—As1	−59.69 (5)	Ru2—P2—C14—C15	−42.34 (19)
Ru1—Ru2—Ru3—As1	−46.38 (3)	C19—C14—C15—C16	0.6 (3)
C48—Ru2—Ru3—Ru1	173.90 (6)	P2—C14—C15—C16	−176.45 (18)
C47—Ru2—Ru3—Ru1	−100.56 (6)	C14—C15—C16—C17	−0.6 (4)
C49—Ru2—Ru3—Ru1	78.91 (6)	C15—C16—C17—C18	0.2 (4)
P2—Ru2—Ru3—Ru1	−13.31 (3)	C16—C17—C18—C19	0.2 (3)
C45—Ru1—Ru3—C51	137.45 (12)	C15—C14—C19—C18	−0.2 (3)
C44—Ru1—Ru3—C51	−142.84 (12)	P2—C14—C19—C18	176.67 (16)
C46—Ru1—Ru3—C51	39.61 (12)	C17—C18—C19—C14	−0.1 (3)
P1—Ru1—Ru3—C51	−73.53 (11)	C14—P2—C20—C25	−105.57 (17)
Ru2—Ru1—Ru3—C51	−35.26 (10)	C13—P2—C20—C25	−1.66 (18)
C45—Ru1—Ru3—C52	66.31 (9)	Ru2—P2—C20—C25	121.60 (15)
C44—Ru1—Ru3—C52	146.02 (9)	C14—P2—C20—C21	76.48 (16)
C46—Ru1—Ru3—C52	−31.53 (8)	C13—P2—C20—C21	−179.60 (14)
P1—Ru1—Ru3—C52	−144.67 (7)	Ru2—P2—C20—C21	−56.35 (16)
Ru2—Ru1—Ru3—C52	−106.40 (6)	C25—C20—C21—C22	−1.0 (3)
C45—Ru1—Ru3—C50	−115.43 (9)	P2—C20—C21—C22	177.00 (15)
C44—Ru1—Ru3—C50	−35.72 (9)	C20—C21—C22—C23	−0.1 (3)
C46—Ru1—Ru3—C50	146.72 (8)	C21—C22—C23—C24	1.1 (3)
P1—Ru1—Ru3—C50	33.59 (7)	C22—C23—C24—C25	−0.9 (3)
Ru2—Ru1—Ru3—C50	71.85 (6)	C21—C20—C25—C24	1.2 (3)
C45—Ru1—Ru3—As1	−17.57 (7)	P2—C20—C25—C24	−176.75 (16)
C44—Ru1—Ru3—As1	62.13 (6)	C23—C24—C25—C20	−0.2 (3)
C46—Ru1—Ru3—As1	−115.42 (6)	C32—As1—C26—C27	−102.95 (17)
P1—Ru1—Ru3—As1	131.45 (2)	C38—As1—C26—C27	153.10 (17)
Ru2—Ru1—Ru3—As1	169.709 (9)	Ru3—As1—C26—C27	23.01 (18)
C45—Ru1—Ru3—Ru2	172.72 (7)	C32—As1—C26—C31	81.46 (17)
C44—Ru1—Ru3—Ru2	−107.58 (6)	C38—As1—C26—C31	−22.49 (18)
C46—Ru1—Ru3—Ru2	74.87 (6)	Ru3—As1—C26—C31	−152.58 (15)
P1—Ru1—Ru3—Ru2	−38.26 (2)	C31—C26—C27—C28	0.7 (3)
C51—Ru3—As1—C32	72.96 (9)	As1—C26—C27—C28	−174.99 (17)
C52—Ru3—As1—C32	165.42 (9)	C26—C27—C28—C29	0.1 (3)
C50—Ru3—As1—C32	−16.55 (9)	C27—C28—C29—C30	−0.5 (3)
Ru2—Ru3—As1—C32	−80.12 (7)	C27—C28—C29—C53	178.3 (2)
Ru1—Ru3—As1—C32	−122.01 (7)	C28—C29—C30—C31	0.1 (3)
C51—Ru3—As1—C38	−165.10 (9)	C53—C29—C30—C31	−178.7 (2)
C52—Ru3—As1—C38	−72.64 (9)	C29—C30—C31—C26	0.7 (3)
C50—Ru3—As1—C38	105.38 (9)	C27—C26—C31—C30	−1.1 (3)
Ru2—Ru3—As1—C38	41.81 (8)	As1—C26—C31—C30	174.55 (16)
Ru1—Ru3—As1—C38	−0.08 (7)	C38—As1—C32—C33	115.60 (17)
C51—Ru3—As1—C26	−47.24 (9)	C26—As1—C32—C33	13.66 (18)
C52—Ru3—As1—C26	45.23 (9)	Ru3—As1—C32—C33	−112.16 (15)
C50—Ru3—As1—C26	−136.75 (9)	C38—As1—C32—C37	−68.31 (18)
Ru2—Ru3—As1—C26	159.68 (7)	C26—As1—C32—C37	−170.24 (17)
Ru1—Ru3—As1—C26	117.79 (6)	Ru3—As1—C32—C37	63.93 (18)
C45—Ru1—P1—C1	76.59 (10)	C37—C32—C33—C34	−1.8 (3)
C44—Ru1—P1—C1	−15.33 (9)	As1—C32—C33—C34	174.32 (16)
C46—Ru1—P1—C1	170.60 (9)	C32—C33—C34—C35	1.3 (3)
Ru2—Ru1—P1—C1	−108.04 (7)	C33—C34—C35—C36	0.3 (4)
Ru3—Ru1—P1—C1	−75.95 (8)	C33—C34—C35—C54	−179.6 (2)
C45—Ru1—P1—C7	−42.07 (9)	C34—C35—C36—C37	−1.4 (4)
C44—Ru1—P1—C7	−134.00 (9)	C54—C35—C36—C37	178.5 (3)
C46—Ru1—P1—C7	51.94 (9)	C35—C36—C37—C32	0.9 (4)
Ru2—Ru1—P1—C7	133.29 (7)	C33—C32—C37—C36	0.7 (3)
Ru3—Ru1—P1—C7	165.39 (7)	As1—C32—C37—C36	−175.50 (19)
C45—Ru1—P1—C13	−164.38 (9)	C32—As1—C38—C39	−13.32 (19)
C44—Ru1—P1—C13	103.69 (9)	C26—As1—C38—C39	92.96 (18)
C46—Ru1—P1—C13	−70.37 (9)	Ru3—As1—C38—C39	−141.50 (15)
Ru2—Ru1—P1—C13	10.98 (7)	C32—As1—C38—C43	172.46 (15)
Ru3—Ru1—P1—C13	43.08 (7)	C26—As1—C38—C43	−81.27 (16)
C48—Ru2—P2—C20	105.96 (9)	Ru3—As1—C38—C43	44.27 (17)
C47—Ru2—P2—C20	15.89 (9)	C43—C38—C39—C40	−0.8 (3)
C49—Ru2—P2—C20	−159.56 (9)	As1—C38—C39—C40	−174.90 (17)
Ru3—Ru2—P2—C20	−66.91 (8)	C38—C39—C40—C41	−0.2 (3)
Ru1—Ru2—P2—C20	−78.58 (7)	C39—C40—C41—C42	1.0 (3)
C48—Ru2—P2—C14	−20.44 (9)	C39—C40—C41—C55	−176.6 (2)
C47—Ru2—P2—C14	−110.51 (9)	C40—C41—C42—C43	−0.8 (3)
C49—Ru2—P2—C14	74.04 (9)	C55—C41—C42—C43	176.8 (2)
Ru3—Ru2—P2—C14	166.69 (7)	C39—C38—C43—C42	0.9 (3)
Ru1—Ru2—P2—C14	155.02 (7)	As1—C38—C43—C42	175.37 (16)
C48—Ru2—P2—C13	−132.77 (9)	C41—C42—C43—C38	−0.2 (3)
C47—Ru2—P2—C13	137.16 (9)	C45—Ru1—C44—O3	−40.5 (9)
C49—Ru2—P2—C13	−38.29 (9)	C46—Ru1—C44—O3	−149.5 (7)
Ru3—Ru2—P2—C13	54.35 (7)	P1—Ru1—C44—O3	54.1 (9)
Ru1—Ru2—P2—C13	42.68 (7)	Ru2—Ru1—C44—O3	146.1 (9)
C7—P1—C1—C2	79.20 (18)	Ru3—Ru1—C44—O3	−159.0 (9)
C13—P1—C1—C2	−175.04 (16)		

### Hydrogen-bond geometry (Å, °)

**Table:**

*D*—H···*A*	*D*—H	H···*A*	*D*···*A*	*D*—H···*A*
C16—H16A···O3^i^	0.93	2.49	3.377 (3)	159
C3—H3A···Cg1^ii^	0.93	2.93	3.708 (3)	142
C22—H22A···Cg2^iii^	0.93	2.78	3.453 (2)	130
C53—H53B···Cg3^iv^	0.96	2.94	3.801 (3)	150

Symmetry codes: (i) −*x*, −*y*, −*z*+1; (ii) *x*+1, *y*, *z*; (iii) *x*−1, *y*, *z*; (iv) −*x*, −*y*+1, −*z*.
